# De novo transcriptome assembly, gene annotation, and EST-SSR marker development of an important medicinal and edible crop, *Amomum tsaoko* (Zingiberaceae)

**DOI:** 10.1186/s12870-022-03827-y

**Published:** 2022-09-29

**Authors:** Mengli Ma, Hengling Meng, En Lei, Tiantao Wang, Wei Zhang, Bingyue Lu

**Affiliations:** 1 Key Laboratory for Research and Utilization of Characteristic Biological Resources in Southern Yunnan, Honghe University, Mengzi, 661199 China; 2 College of Biological and Agricultural Sciences, Honghe University, Mengzi, 661199 China

**Keywords:** *Amomum tsaoko*, Transcriptome sequencing, EST-SSR markers, Genetic diversity

## Abstract

**Background:**

*Amomum tsaoko* is a medicinal and food dual-use crop that belongs to the Zingiberaceae family. However, the lack of transcriptomic and genomic information has limited the understanding of the genetic basis of this species. Here, we performed transcriptome sequencing of samples from different *A. tsaoko* tissues, and identified and characterized the expressed sequence tag-simple sequence repeat (EST-SSR) markers.

**Results:**

A total of 58,278,226 high-quality clean reads were obtained and de novo assembled to generate 146,911 unigenes with an N50 length of 2002 bp. A total of 128,174 unigenes were successfully annotated by searching seven protein databases, and 496 unigenes were identified as annotated as putative terpenoid biosynthesis-related genes. Furthermore, a total of 55,590 EST-SSR loci were detected, and 42,333 primer pairs were successfully designed. We randomly selected 80 primer pairs to validate their polymorphism in *A. tsaoko*; 18 of these primer pairs produced distinct, clear, and reproducible polymorphisms. A total of 98 bands and 96 polymorphic bands were amplified by 18 pairs of EST-SSR primers for the 72 *A. tsaoko* accessions. The Shannon's information index (I) ranged from 0.477 (AM208) to 1.701 (AM242) with an average of 1.183, and the polymorphism information content (PIC) ranged from 0.223 (AM208) to 0.779 (AM247) with an average of 0.580, indicating that these markers had a high level of polymorphism. Analysis of molecular variance (AMOVA) indicated relatively low genetic differentiation among the six *A. tsaoko* populations. Cross-species amplification showed that 14 of the 18 EST-SSR primer pairs have transferability between 11 Zingiberaceae species.

**Conclusions:**

Our study is the first to provide transcriptome data of this important medicinal and edible crop, and these newly developed EST-SSR markers are a very efficient tool for germplasm evaluation, genetic diversity, and molecular marker-assisted selection in *A. tsaoko*.

**Supplementary Information:**

The online version contains supplementary material available at 10.1186/s12870-022-03827-y.

## Background

*Amomum tsaoko* is a perennial evergreen tufted herb of Zingiberaceae; the whole plant has a spicy taste. The dried fruit (also called Cao-Guo in China) of *A. tsaoko* is an important crude drug in traditional Chinese medicine (TCM), such as ‘Cao-Guo-Zhi-Mu-Tang,’ ‘Da-Yuan-Yin,’ ‘Cao-Guo-Si-Wei-Tang,’ and ‘Li-Gan-Shi-Liu-Ba-Wei-San,’ which clear dampness, resolve phlegm, warm the spleen, and dispel colds. In addition, *A. tsaoko* is also a top-grade spice known as one of the “five spices” in food seasoning [1]. In recent years, *A. tsaoko* has been considered to have a broader utilization value and proven to have biological activities, such as anti-oxidation [2, 3], antibacterial [4], anti-inflammation [5], antidiabetic [6], anti-tumor [7], and anticonvulsant properties [8]. In addition, *A. tsaoko* is a TCM that has been prescribed for the treatment of COVID-19 [9–12]. As an aromatic Chinese herbal medicine used for both medicinal and edible purposes, essential oil is the most important active ingredient of *A. tsaoko*, and its content determines the quality of *A. tsaoko* (Pharmacopoeia of the People’s Republic of China, 2020). Recent studies have shown that *A. tsaoko* essential oil has a significant inhibitory effect on COVID-19 [13]. The monoterpene 1,8-cineole was found to be the major constituent (34.6–45.24%) of the essential oil in *A. tsaoko*, and has well-known antiviral, anti-inflammatory, antimicrobial, and pain-relieving effects [14]. In recent years, the cloning and expression analysis of functional genes involved in terpenoid biosynthesis has become a popular topic of research. He et al. [15] used transcriptome sequencing to identify a number of terpenoid synthesis-related genes in *Amomum villosum*, including five monoterpene synthase genes *AvTPS1*–*AvTPS5*. However, until now, there have been no reports about regulated genes that are involved in the terpenoid biosynthesis of *A. tsaoko*.

*A. tsaoko* is a high-altitude medicinal crop that grows in humid forests, narrowly distributed in the southern Yunnan Province of China and northern Laos and Vietnam at high altitudes between 1100 and 1800 m in mountainous regions [16, 17]. Due to excessive harvesting and the destruction of the original habitat of *A. tsaoko*, wild resources are almost extinct, and it was listed as a “Nearly Endangered Species” on the IUCN Red List in 2012. Genetic diversity evaluation can provide important reference information for the identification and evaluation of germplasm resources and the selection of elite germplasm. Molecular marker technology is an effective tool for analyzing plant genetic diversity, which has the advantages of abundant quantity, high polymorphism, direct expression in the form of DNA, and is not affected by the environment [18, 19]. SSRs, also known as microsatellite DNA, are short, repeated DNA sequences present throughout the genome. It has the advantages of good reproducibility, codominance, abundant polymorphisms, and easy detection [20]. In recent years, SSR markers have been widely used in genetic diversity analysis [21, 22], linkage genetic map construction and QTL identification [23, 24], and marker-assisted breeding [25, 26].

As a TCM and spice, previous studies of *A. tsaoko* mainly focused on active component extraction, identification of chemical components, and pharmacological effects [27–32]; however, molecular studies of *A. tsaoko* are lacking. This limits the excellent germplasm selection and breeding utilization of *A. tsaoko*. Some researchers have begun to study the genetic diversity of *A. tsaoko* at the phenotypic and molecular levels. Zhang et al. [33] analyzed the phenotypic characteristics of *A. tsaoko* in nine producing areas of China. The results showed that the number of fruit ridges, the number of seeds per fruit, and the vertical diameter of fruit had the greatest variation. Genetic diversity and population genetic differentiation of *A. tsaoko* from eight different producing areas were analyzed by 12 RAPD markers [34]. More recently, the genetic diversity of 91 *A. tsaoko* accessions from southwest China was studied by SRAP and ISSR markers [17]. A few reports on SSR development and the genetic diversity of *A. tsaoko* have recently been published [1, 35], but the number of markers is insufficient to conduct comprehensive genetic studies.

With the rapid development of next-generation sequencing technology and lower sequencing costs, large-scale RNA-seq provides an important information for functional gene mining and molecular marker development in non-model species [36, 37]. Here, we present the first transcriptome of *A. tsaoko* using the Illumina HiSeqTM 4000 sequencing platform. The obtained transcriptome data advance our understanding of the function categories from the annotated genes on this species, and the development of EST-SSR markers will provide an important basis for germplasm evaluation, genetic diversity analysis, and molecular breeding of *A. tsaoko*.

## Results

### Sequencing and de novo assembly

In this study, 59,876,622 raw reads were obtained using the Illumina HiSeq 4000 platform. After strict quality control, 97.33% clean reads (58,278,226) were obtained with 96.28% Q20 and 94.25% Q30 bases. A total of 199,191 transcripts were obtained by de novo assembly of clean reads with Trinity software. After clustering transcripts and removing redundancy, 146,911 unigenes were obtained, with an average length of 1527 bp and an N50 value of 2002 bp (Table 1).

**Table 1:** Characterization of *Amomum tsaoko* transcripts

Category	Item	Number
Sequenced reads	raw reads	59,876,622
	clean reads	58,278,226
	clean bases	8.74G
	error (%)	0.03
	Q20 (%)	96.28
	Q30 (%)	94.25
	GC content (%)	50.22
Transcripts	total number	199,191
	minimum length (bp)	201
	mean length (bp)	1219
	maximum length (bp)	10,949
	N50	1888
	N90	571
	total nucleotides	242,805,690
Unigenes	total number	146,911
	minimum length (bp)	201
	mean length (bp)	1527
	maximum length (bp)	10,949
	N50	2002
	N90	801
	total nucleotides	224,308,460

**Fig. 1:**
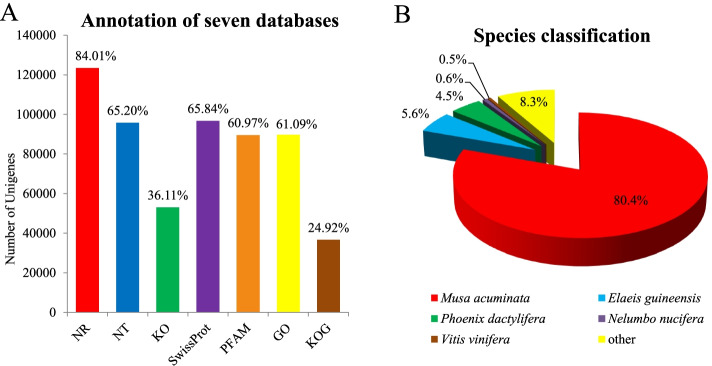
Functional annotation (**A**) and species distribution (**B**) of unigenes in *Amomum tsaoko*

The assembled unigenes of *A. tsaoko* were functionally annotated against seven public databases (Table 2, Fig. 1A). A total of 123,420 unigenes (84.01%) were annotated successfully in the NR database, while 95,786 (65.20%) were annotated in the NT database. At the same time, 21,543 unigenes were annotated in all databases, and 128,174 unigenes (87.24%) were successfully annotated with at least one database. The species distribution showed that 80.4% matched *Musa acuminata*, and 5.6% matched *Elaeis guineensis*. The matching degrees of *Phoenix dactylifera*, *Nelumbo nucifera*, and *Vitis vinifera* were 4.5, 0.6, and 0.5%, respectively, and 8.3% for other species (Fig. 1B).

**Table 2:** Functional annotation of *A. tsaoko* in seven databases

Annotation database	Number of unigenes	Percentage (%)
Annotated in NR	123,420	84.01
Annotated in NT	95,786	65.20
Annotated in KEGG	53,059	36.11
Annotated in SwissProt	96,740	65.84
Annotated in PFAM	89,573	60.97
Annotated in GO	89,750	61.09
Annotated in KOG	36,621	24.92
Annotated in all databases	21,543	14.66
Annotated in at least one database	128,174	87.24
Total unigenes	146,911	100

**Fig. 2:**
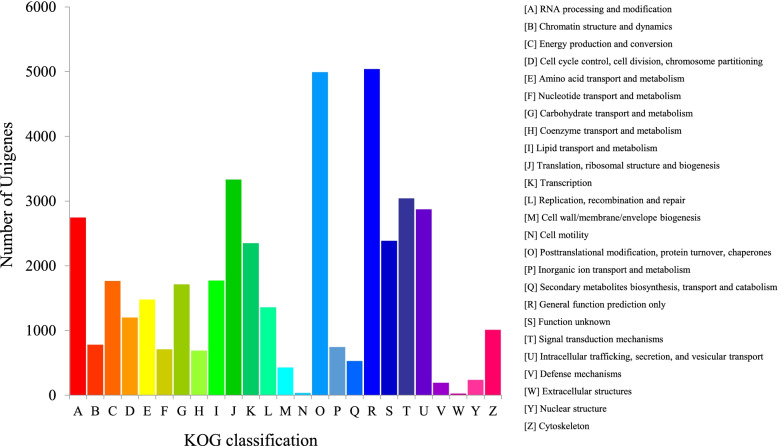
Eukaryotic clusters of orthologous groups (KOG) classification of unigenes. A total of 36,621 unigenes with NR hits were grouped into 25 functional groups. The *y*-axis indicates the number of unigenes in a specific functional cluster. The *x*-axis indicates the function class

**Fig. 3:**
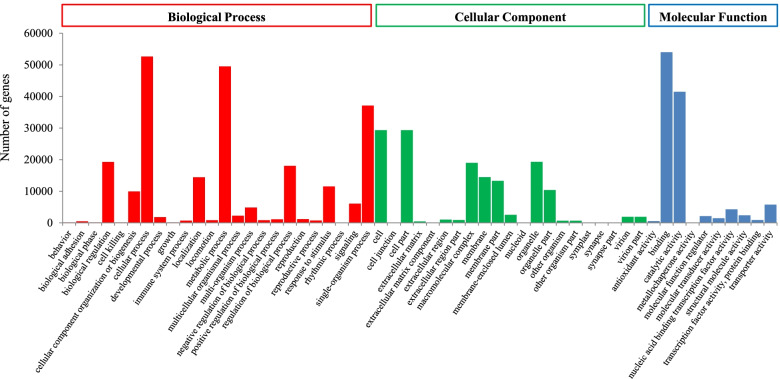
Gene ontology (GO) classification of unigenes. A total of 89,750 unigenes were categorized into three main categories: biological process, cellular component, and molecular function. The *x*-axis indicates the subgroups in GO annotation, while the *y*-axis indicates the percentage of specific categories of genes in each main category

**Fig. 4:**
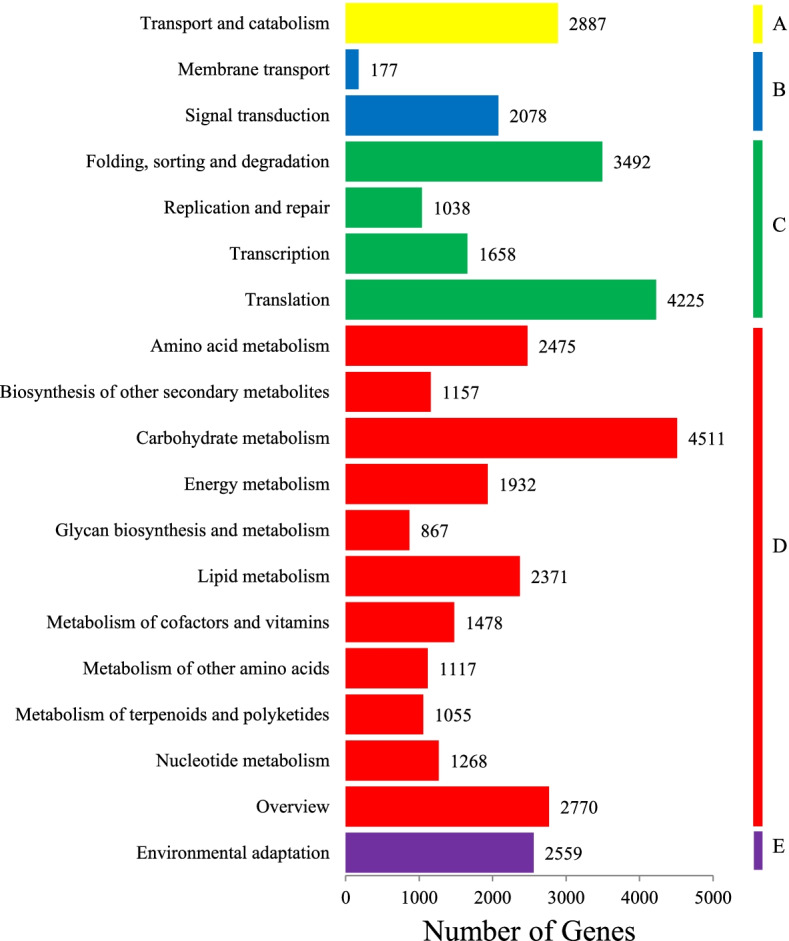
Clusters of orthologous groups based on KEGG classification. **A** Cellular process; **B** Environmental information processing; **C** Genetic information processing; **D** Metabolism; **E** Organismal systems

Furthermore, for the KOG classification, 36,621 putative unigenes of *A. tsaoko* were classified into 25 clusters (Fig. 2). Among these categories, ‘general function prediction only’ (5039; 13.76%); ‘posttranslational modification, protein turnover, chaperones’ (4993; 13.63%); and ‘translation, ribosomal structure and biogenesis’ (3332; 9.10%) were the dominant groups, while only a few unigenes were annotated as ‘cell motility’ (35; 0.09%) and ‘extracellular structures’ (26; 0.07%). Furthermore, there were 89,750 unigenes categorized into three main GO categories: biological processes (233,859; 47.53%); cellular components (145,239; 29.52%); and molecular functions (112,917; 22.95%) (Fig. 3). Within the three categories, ‘binding’ (53,980), ‘cellular process’ (52,634), ‘metabolic process’ (49,492), ‘catalytic activity’ (41,468), and ‘single-organism process’ (37,122) were the most prevalent. Following searches against the KEGG database, 53,059 unigenes were classified into five categories, which were distributed in 130 metabolic pathways. The largest category was comprised of ‘metabolism’ (21,001; 53.69%), followed by ‘genetic information processing’ (10,413; 26.62%), ‘cellular processes’ (2887, 7.38%), ‘organismal systems’ (2559, 6.54%), and ‘environmental information processing’ (2255, 5.77%) (Fig. 4). In addition, 96,740 (65.84%) and 89,573 (60.97%) unigenes matched the SwissProt and PFAM databases, respectively.

**Fig. 5:**
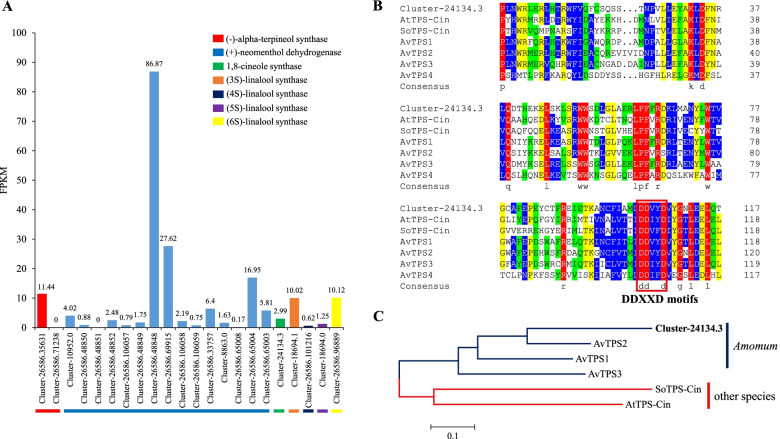
Unigene analysis of potential monoterpene metabolic pathway in *A. tsaoko*. Expression of candidate unigenes related to monoterpene synthesis of *A. tsaoko* (**A**). Alignment of amino acid sequences of Cluster-24,134.3 (putative 1,8-cineole synthase unigene) with other TPS proteins from *Arabidopsis thaliala* (AtTPS-Cin), *Salvia officinalis* (SoTPS-Cin), and *Amomum villosum* (AvTPS1-AvTPS4) using DNAMAN 8.0 (**B**). Phylogenetic tree constructed based on amino acid multiple sequence alignment using MEGA 5.2 with the neighbor-joining method (**C**), dark blue branches represent genus *Amomum* species (*A. tsaoko* and *A. villosum*), and red branches represent other species (*A. thaliala* and *S. officinalis*)

Importantly, KEGG analysis showed that 496 unigenes were annotated as being directly involved in the metabolism of terpenoids, including the terpenoid backbone (338 unigenes), monoterpenoid (22 unigenes), diterpenoid (81 unigenes), and sesquiterpenoid and triterpenoid (55 unigenes) biosynthesis pathways (Table 3). In the monoterpenoid biosynthesis pathway, two unigenes encoding alpha-terpineol synthase, 15 unigenes encoding neomenthol dehydrogenase, one unigene encoding 1,8-cineole synthase, and four unigenes encoding linalool synthase were significantly enriched. The FPKM values of Cluster-26,586.35631, Cluster-26,586.48848, Cluster-26,586.69915, Cluster-26,586.65004, Cluster-18,694.1, and Cluster-26,586.96889 were greater than 10 (Fig. 5A). Notably, 1,8-cineole synthase (Cluster-24,134.3) was initially identified in this species. Multiple sequence alignment revealed that Cluster-24,134.3 contained the DDXXD motif, which is conserved in angiosperm monoterpene synthases (Fig. 5B). Cluster analysis showed that Cluster-24,134.3 had the highest homology with the monoterpene synthase gene *AvTPS2* in *Amomum villosum* (Fig. 5C).

**Table 3:** Overview of the terpene biosynthetic pathways

Pathway	No. of unigenes	Pathway ID
Terpenoid backbone biosynthesis	338	ko00900
Monoterpenoid biosynthesis	22	ko00902
Diterpenoid biosynthesis	81	ko00904
Sesquiterpenoid and triterpenoid biosynthesis	55	ko00909

### Development of EST-SSR markers

**Fig. 6:**
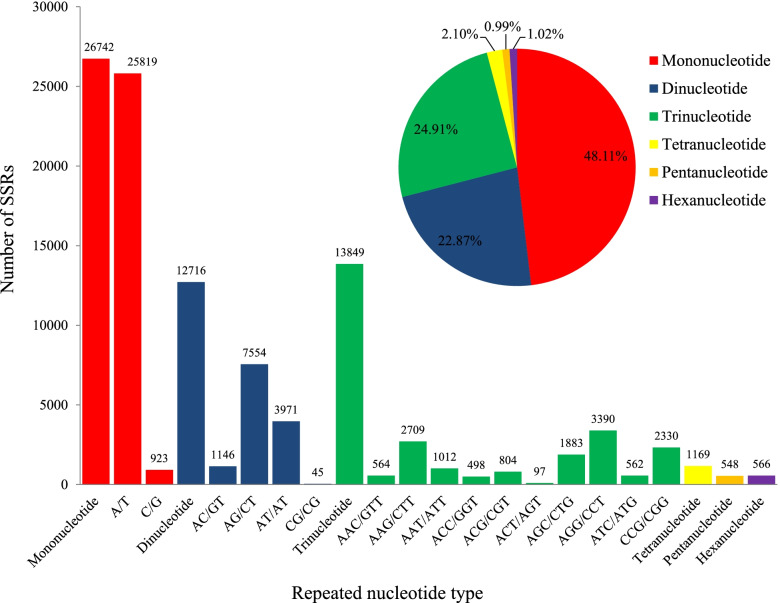
Frequency and distribution of EST-SSRs from the transcriptome of *A. tsaoko.* The most abundant dinucleotide and trinucleotide motifs were AG/CT and AGG/CCT

SSR loci were identified within the *A. tsaoko* transcriptome using the MISA software. Among the 146,911 non-redundant unigenes, we identified 55,590 potential SSRs, of which 3794 had a compound formation. There were 10,465 unigenes containing more than one SSR. An average of one SSR site was found every 1.53 kb. The largest fraction of SSRs were mononucleotide SSRs (26, 742, 48.11%), followed by trinucleotide repeats (13,849; 24.91%), dinucleotide repeats (12,716; 22.87%), tetranucleotide repeats (1169; 2.10%), hexanucleotide repeats (566; 1.02%), and pentanucleotide repeats (548; 0.99%) (Fig. 6, Table 4). Among the different SSR repeat-type classes, the most dominant repeat motifs were A/T (25,819; 46.44%), AG/CT (7554; 13.59%), AT/AT (3971; 7.14%), AGG/CCT (3390; 6.10%), AAG/CTT (2709; 4.87%), and CCG/CGG (2330; 4.19%). The remaining motif types accounted for 17.67% of these repeats (Table 4). The tandem repeat numbers of these SSRs ranged from 5 to 86, and 10 tandem repeats (12,910; 23.22%) was the most common number, followed by 5 (7789; 14.01%), 6 (6749; 12.14%), 11 (5655; 10.17%), 12 (3975; 7.15%), and 7 (3792; 6.82%) (Table 5).

**Table 4:** Distribution of EST-SSR loci in the transcriptome of *A. tsaoko*

Repeat unit type	Motif	SSR number	Ratio (%)	Dominating motif		
				Motif type	Number	Ratio (%)
Mononucleotide	2	26,742	48.11	A/T	25,819	96.55
Dinucleotide	4	12,716	22.87	AG/CT	7554	59.41
Trinucleotide	10	13,849	24.91	AGG/CCT	3390	24.48
Tetranucleotide	30	1169	2.10	AAAG/CTTT	197	16.85
Pentanucleotide	58	548	0.99	AAAAT/ATTTT	66	12.04
Hexanucleotide	72	566	1.02	AGGCGG/CCGCCT	42	7.42
Total	176	55,590

**Table 5:** The length distribution of EST-SSRs based on the number of nucleotide repeat units

Repeats	Repetition times										
	5	6	7	8	9	10	11	12	13	14	≥15
Mononucleotide						11,545	4455	2905	1896	1370	4571
Dinucleotide		3002	1961	1433	1132	982	921	948	521	426	1390
Trinucleotide	6330	3207	1694	1185	442	371	275	116	67	43	119
Tetranucleotide	754	310	52	31	6	4	1	6	3		2
Pentanucleotide	385	101	33	20	5	4					
Hexanucleotide	320	129	52	36	20	4	3		1		1
Total	7789	6749	3792	2705	1605	12,910	5655	3975	2488	1839	6083
Distribution frequency (%)	14.01	12.14	6.82	4.87	2.89	23.22	10.17	7.15	4.48	3.31	10.94

### Validation of EST-SSR markers

**Fig. 7:**
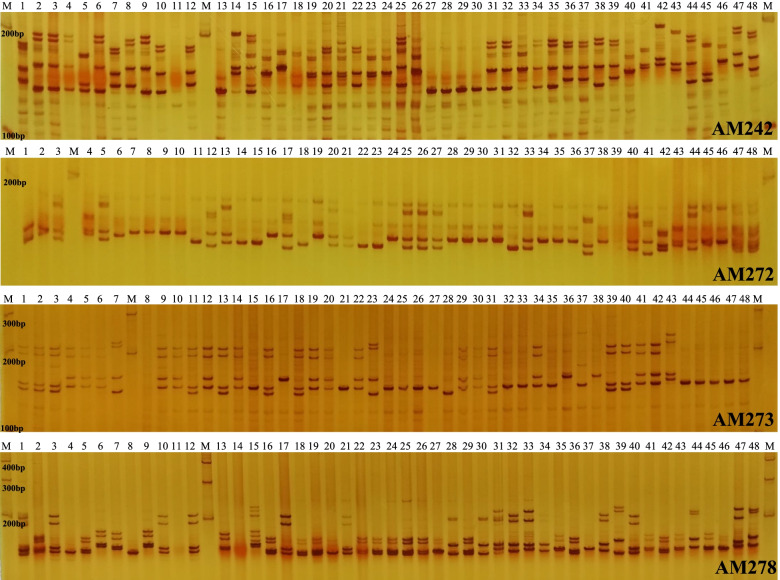
Electrophoretic profiles of 48 *A. tsaoko* accessions using four pairs of EST-SSR primers. Denatured PCR products were separated using an 8% non-denaturing polyacrylamide gel and then stained with 1% silver nitrate

A total of 42,333 primer pairs were successfully designed based on SSR flanking sequences. Four accessions of *A. tsaoko* were used for primer amplification specificity and efficiency testing. Eighty pairs of primers were randomly selected for synthesis; 64 of the EST-SSRs successfully amplified DNA fragments with the expected band sizes. Eventually, 18 primer pairs yielding the best amplification were chosen to analyze the genetic diversity of 72 *A. tsaoko* accessions from six cultivated populations (Table 6). SSR amplification profiles of primer pairs AM242, AM272, AM273, and AM278 are shown in Fig. 7. A total of 98 bands were scored, with an average of 5.4 bands per primer and a range of 3 (AM213) to 12 (AM255). The average effective number (Ne) of alleles per locus was 2.961, ranging from 1.311 (AM208) to 4.852 (AM242). The Shannon' information index (I) ranged from 0.477 (AM208) to 1.701 (AM242) with a mean of 1.183. The observed heterozygosity (Ho) ranged from 0.264 (AM208) to 1.000 (AM225) with a mean of 0.594, and the expected heterozygosity (He) ranged from 0.237 (AM208) to 0.794 (AM242) with a mean of 0.613. The value of PIC ranged from 0.223 (AM208) to 0.779 (AM247) with a mean value of 0.580. The HWE analysis showed that six loci (AM224, AM225, AM237, AM247, AM248, and AM278) significantly deviated from HWE (*P* < 0.05), and the remaining 12 loci (AM203, AM206, AM207, AM208, AM213, AM218, AM223, AM242, AM255, AM272, AM273, and AM279) were in accordance with HWE (Table 7). The frequencies of null alleles were low (< 0.20) for each locus, except AM247.

**Table 6:** Characterization of 18 EST-SSRs among 72 *A. tsaoko* accessions

Locus	Primer sequences (5′–3′)	Repeat motif	Ta (°C)	Size (bp)	NB	NPB	PPB
AM203	F: TTCAACCCCACGACACACAA	(AAGGCG)5	55	156	5	5	100.00%
	R: AGGATGAAGGCTGAGGAGGT						
AM206	F: ACAGAGAACCACAGGCGAAG	(AGA)13	55	164	7	7	100.00%
	R: GACGAGTTGGGAAGGGATGG						
AM207	F: GAGCCGATCGAGCTAACGAA	(AGAA)5	55	168	5	4	80.00%
	R: TGGAAAGGTAGGTGCAACGT						
AM208	F: CGCACCCCAATTCTCCTTCT	(AGC)7	55	160	4	3	75.00%
	R: GCGGATCAGTATCATCCCCG						
AM213	F: GTTTCCGCCGTGAACTAACG	(ATC)5	55	167	3	3	100.00%
	R: TATGGCCTAGTCACGTTGGC						
AM218	F: GCTTCCTCTCCTATGACGCC	(CCGCCT)6	55	159	5	5	100.00%
	R: TGCTTTCGTCGCTTGTCTCT						
AM223	F: TGTTCTTCTACTGCTGCGCT	(CCTCT)6	55	166	5	5	100.00%
	R: TGGTGTGAAGGAGAGGAGGA						
AM224	F: TTGCTAGAGCTCAAGCCACC	(CGA)9	55	170	6	6	100.00%
	R: CAAAGCTCGAGGATAGGCGT						
AM225	F: TCGTGATCCCTTCGCTTTGT	(CGAAGG)5	55	166	6	6	100.00%
	R: GCTCCATCGCCTCCAACATA						
AM237	F: GTGTGATGGGGGTAAGGGTG	(GAA)8	55	158	4	4	100.00%
	R: AGCGCCGTAAAAGGTCCTTT						
AM242	F: GAGAGCGAGTTGAGCCATGA	(GCA)12	55	166	7	7	100.00%
	R: GCCGGTGACAAAGATGGAGA						
AM247	F: AAGAGAGCATCAAGAGGCCG	(GGA)10	55	163	6	6	100.00%
	R: CGACCGAACCTTGTAACCCA						
AM248	F: CGTCTAGTGCTCCGGAATCC	(GGA)6	55	157	4	4	100.00%
	R: GCCATAGCTCCCTCTCCCTT						
AM255	F: CGGGAACAACGACGGTAAGA	(GGCGGA)7	55	166	12	12	100.00%
	R: CGGAATCACAATCGCCATCG						
AM272	F: GCGATCTCCAGGGCGAAATA	(TGC)7	55	156	4	4	100.00%
	R: TTTAGCCCTCCTCCTCCTCC						
AM273	F: AAAGAAGGAATCTGGCCCGG	(TGCCTC)6	55	164	5	5	100.00%
	R: ACGGCGAAAGGTCAGCAATA						
AM278	F: TGCCTGCGCTTAGTCAATCA	(TTC)7	55	157	6	6	100.00%
	R: CCCTCCAATGTTCCCAACGA						
AM279	F: ATTGCAATTGAAGCAGCGCA	(TTCT)6	55	156	4	4	100.00%
	R: GGTGGTTTGCATCCATGGTG						

*Ta* annealing temperature, *NB* Number of amplified bands, *NPB* Number of polymorphic bands; *PPB* Percentage of polymorphic bands

**Table 7:** Allelic diversity of 18 EST-SSR markers used in 72 accessions of *A. tsaoko*

Locus	Ne	I	Ho	He	PIC	HWE	FNA
AM203	3.014	1.171	0.653	0.668	0.600	0.962	0.018
AM206	3.977	1.529	0.743	0.749	0.723	0.520	−0.067
AM207	1.371	0.532	0.310	0.271	0.270	0.881	−0.026
AM208	1.311	0.477	0.264	0.237	0.223	0.645	−0.049
AM213	2.127	0.830	0.543	0.530	0.463	0.583	0.038
AM218	2.293	0.963	0.592	0.564	0.490	0.973	−0.054
AM223	3.187	1.298	0.704	0.686	0.643	0.776	0.178
AM224	2.668	1.274	0.606	0.625	0.599	0.024*	−0.034
AM225	3.452	1.423	1.000	0.710	0.684	0.000***	−0.046
AM237	2.741	1.121	0.571	0.635	0.591	0.006**	0.066
AM242	4.852	1.701	0.775	0.794	0.771	0.177	−0.062
AM247	4.393	1.638	0.348	0.772	0.779	0.000***	0.335
AM248	2.814	1.169	0.614	0.645	0.610	0.003**	−0.046
AM255	4.001	1.686	0.689	0.750	0.770	0.957	−0.111
AM272	2.331	1.065	0.507	0.571	0.538	0.286	0.051
AM273	2.726	1.114	0.681	0.633	0.563	0.692	0.032
AM278	4.263	1.576	0.704	0.765	0.738	0.001**	0.034
AM279	1.771	0.733	0.394	0.435	0.386	0.761	−0.058
Mean	2.961	1.183	0.594	0.613	0.580	0.458	0.011

*Ne* effective number of alleles, *I* Shannon’s information index, *Ho* observed heterozygosity, *He* expected heterozygosity, *PIC* Polymorphic information content, *HWE* Hardy–Weinberg equilibrium, *FNA* Frequencies of null alleles. Significant deviation from HWE at * *P* < 0.05, ** *P* < 0.01, and *** *P* < 0.001

### Population genetic diversity

**Fig. 8:**
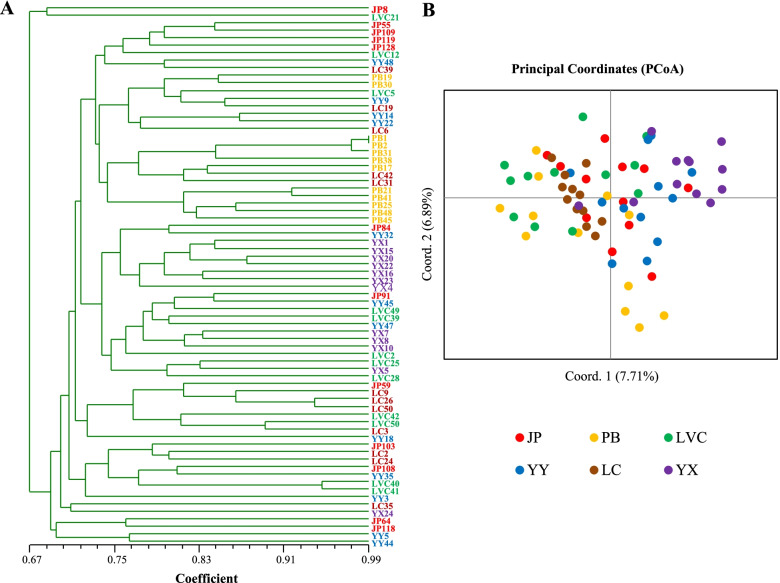
Cluster analysis and principal coordinate analysis (PCoA) based on the genotyping of 72 accessions of *A. tsaoko*

At the population level, the YY population scored higher values in Ne, I, and He, while Na and Ho were higher in the LVC and LC populations, respectively. Notably, the number of private alleles (PAr) in the JP and LVC populations (0.167) was higher than that in the PB, YY, LC, and YX populations. Mean fixation index (F) values ranged from − 0.145 in the YX population to 0.015 in the YY population, with an average of − 0.066 (Table 8). The samples did not cluster according to the six different sampling sites based on the UPGMA cluster and PCoA, and all samples from different populations were not separated from each other (Fig. 8). The studied populations showed relatively low between-population genetic differentiation (Fst average = 0.052; min = 0.029, max = 0.091) and small genetic distance (GD average = 0.155, min = 0.090, max = 0.263) (Table 9). Consistent with these results, AMOVA also showed that 90% molecular variance was found within populations (Table 10).

**Table 8:** Genetic diversity of *A. tsaoko* populations based on EST-SSR markers

Population	PPB/%	Na	Ne	I	Ho	He	PAr	F
JP	100.000	4.000	2.662	1.078	0.573	0.582	0.167	− 0.009
PB	88.890	3.167	2.481	0.927	0.596	0.535	0.056	−0.110
LVC	100.000	4.111	2.714	1.073	0.584	0.578	0.167	−0.009
YY	100.000	4.000	2.747	1.086	0.576	0.591	0.056	0.015
LC	100.000	3.722	2.450	0.981	0.620	0.540	0.111	−0.140
YX	100.000	3.722	2.415	0.967	0.617	0.537	0.111	−0.145
Mean	98.150	3.787	2.578	1.019	0.594	0.560	0.111	−0.066

*PPB* Percentage of polymorphic bands, *Na* observed number of alleles, *Ne* effective number of alleles, *I* Shannon’s information index, *Ho* observed heterozygosity, *He* expected heterozygosity, *PAr* number of private alleles, *F* Fixation index

**Table 9:** Pairwise comparison of F_ST_ (above diagonal) and genetic distance (below diagonal) for six *A. tsaoko* populations

Population	JP	PB	LVC	YY	LC	YX
JP	–	0.058	0.029	0.030	0.051	0.057
PB	0.166	–	0.050	0.058	0.059	0.091
LVC	0.090	0.144	–	0.040	0.041	0.069
YY	0.103	0.175	0.132	–	0.041	0.044
LC	0.147	0.156	0.115	0.127	–	0.068
YX	0.170	0.263	0.204	0.130	0.207	–

**Table 10:** Analysis of molecular variance (AMOVA) in *A. tsaoko* from six populations

Source of variation	df	Sum of squares	Variance components	Total variation (%)	*P* value
Among populations	5	65.535	0.306	5%	
Among individuals within populations	66	380.708	0.283	5%	
Within individuals	72	374.500	5.201	90%	< 0.001
Total	143	820.743	5.791		

### Cross-species transferability

**Fig. 9:**
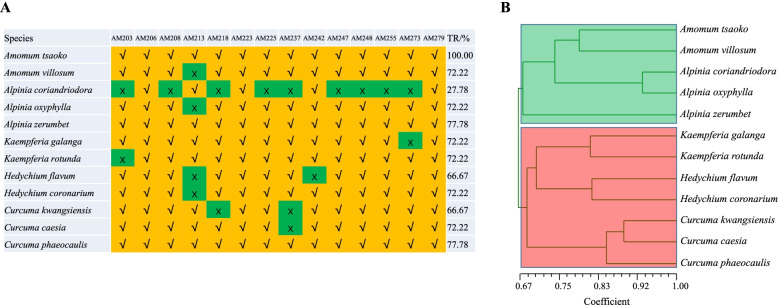
Cross-species transferability of 14 polymorphic EST-SSR markers in different Zingiberaceae species. The transferability rates of EST-SSR markers in 11 Zingiberaceae species (**A**). UPGMA phylogenetic analysis of 12 Zingiberaceae species based on transferable SSR markers (**B**)

To test the transferability of the novel EST-SSR markers in different Zingiberaceae species, 18 polymorphic EST-SSR markers were tested for amplification in 11 other species. Fourteen primer pairs were successfully amplified, and the transferability rates (TR) ranged from 27.78% (*Alpinia coriandriodora*) to 77.78% (*Alpinia zerumbet* and *Curcuma phaeocaulis*), with an average of 68.18% (Fig. 9A). Furthermore, UPGMA phylogenetic analysis based on transferable SSR markers showed that 12 Zingiberaceae species clustered into two groups (Fig. 9B): the first class (the green shaded area indicating the genera *Amomum* and *Alpinia*) and the second class (the red shaded area indicating the genera *Kaempferia*, *Hedychium*, and *Curcuma*).

## Discussion

### Characterization of the *Amomum tsaoko* transcriptome

For species without reference genomes, transcriptome sequencing is considered the most effective way of mining functional genes and developing novel molecular markers [38, 39]. The results of this study represent the first report of transcriptome analysis and EST-SSR detection in *A. tsaoko*. To maximize the transcriptome information gained, mixed samples of five organs, namely roots, stem, leaves, flowers, and fruit, were collected from *A. tsaoko* for paired-end transcriptome sequencing. A total of 58,278,226 clean and high-quality reads with a 94.25% Q30 level, which ensures the quality of sequencing. In the present study, the N50 sizes of generated unigenes (2002 bp) were obviously longer than those in the Zingiberaceae family, such as *A. villosum* (N50 = 1381 bp) [15], *C. wenyujin* (N50 = 1566 bp) [40], *Curcuma longa* (N50 = 1515 bp) [41], *Curcuma alismatifolia* (N50 = 1501 bp) [42], *Zingiber officinale* (N50 = 1077 bp) [43], and *Elettaria cardamomum* (N50 = 616–664 bp) [44].

Among them, 128,174 unigenes were annotated to 7 major databases, such as GO, KEGG, NR, and KOG, accounting for 87.24% of the total unigenes. These annotated unigenes provide a reference basis for the study of metabolic pathways, gene function classification, plant hormone signal transduction, and quality character analysis of *A. tsaoko*. In addition, there are 18,737 unigenes unannotated. It is speculated that these unigenes may be specific new genes of *A. tsaoko* or that the non-coding RNA sequence and public gene database are imperfect [45–47]. Based on NR alignment results, most of the unigenes were annotated to *Musa acuminata* (80.4% similarity), similar to the report in *Curcuma alismatifolia* (Zingiberaceae family) (80.4% similarity) [42]. Zingiberaceae (*A. tsaoko*) and Musaceae (*Musa acuminata*) belong to Zingiberacea, and both are tropical plants, which may explain the large number of homologous genes between them [48]. In addition, 8.3% of the homologous sequences have not been matched, which may because some unigene fragments are too small to match a single data sequence. *A. tsaoko* can be divided into 3 major categories and 55 subcategories in GO functional classification, mainly focusing on cellular part, catalytic activity, and metabolic process. The same outcome was found in the transcriptomic study of *A. villosum* from congeneric species [15]. Volatile terpenoids are the active metabolites in the essential oil of *A. tsaoko*, but the terpene synthases (TPS) responsible for their biosynthesis remain unknown. In our study, the KEGG enrichment analysis found that 496 unigenes were enriched in the metabolic pathways of terpenoids, which will provide a better understanding of terpenoid biosynthesis in *A. tsaoko*. The DDXXD conserved motifs contained in the amino acid sequence of Cluster-24,134.3 binds catalytic substrates (such as GPP) through complexation with metal ions (such as Mg^2+^), which is shared by ionization-dependent terpenoid synthases belonging to the monoterpene synthase functional domain [49, 50]. The phylogenetic tree based on amino acid sequences showed that Cluster-24,134.3 had the highest homology with the AvTPS2 identified in *A. villosum*, which is in the same genus, and was quite different from the 1,8-cineole synthase gene of *Arabidopsis* and *Salvia officinalis*. This may be because the phylogeny and lineage differentiation of angiosperm TPS are closely related to the natural plant classification system, and the similarity of TPS from similar species is much higher than that of TPS from different sources with the same function [51]. Follow-up cloning and functional verification of the corresponding genes of Cluster-24,134.3 are necessary.

### Frequency and distribution of EST-SSRs

Molecular marker technology has been widely used in various fields of plant sciences, including germplasm resource identification, genetic diversity, new variety breeding, and map construction [18]. Among them, SSRs are known as a marker type widely used at present. Furthermore, SSRs can be divided into two categories: genomic and expressed sequence tags (EST-SSRs). The construction of a transcriptome platform has promoted the development of DNA molecular markers to a great extent. To date, there are only a few reports about molecular markers in *A. tsaoko* [1, 17, 35]. Recently, 123 SSRs were identified in the *A. tsaoko* chloroplast genome; the mean density of SSR loci is up to 1/1.33 kb; however, they have not been validated for *A. tsaoko* [48]. In recent years, the use of transcriptome data to obtain sequences containing microsatellites, and the study of their genetic diversity has been successfully reported [52–54]. Excluding mononucleotide repeats, trinucleotides were found to be the most abundant repeats in the present study. This result was consistent with previous studies in other plants, such as *Curcuma alismatifolia* [42], *Vicia amoena* [22], and *Pseudotaxus chienii* [55]. Importantly, the abundance of trinucleotides in coding regions does not change the coding frame and therefore may not affect the functions of the genes [56, 57]. The most abundant dinucleotide SSR motifs were AG/CT (59.41%), a similar finding has been reported in other plant genomes [42, 53, 58, 59]. This may be because alanine (Ala) and leucine (Leu) are the amino acids with the highest frequency in proteins, while the AG/CT dinucleotide motif is present in the codons of Ala and Leu [60]. Moreover, some studies have shown that the CT motif frequently occurs in the 5′ UTRs; this motif plays an important role in regulating nucleic acid metabolism and gene expression in plants [61, 62]. Among trinucleotide repeat motifs, AGG/CCT (24.48%) occurred most commonly, agreeing with the results reported in *Zantedeschia rehmannii* [63], *Phragmites karka* [64], *Amorphophallus* [65], and *Aspidistra saxicola* [66]. It has been suggested that AGG/CCT interruptions play a protective role in genomic packaging [67, 68].

### Validation of EST-SSR markers

In our study, 64 primer pairs (80%) were successfully amplified among the 80 randomly selected EST-SSRs (designated AM201-AM280) across 4 *A. tsaoko* accessions. Some studies have pointed out that, compared with genomic SSRs, EST-SSRs have a better amplification effect, but the polymorphism rate is relatively low [69, 70]. However, these newly developed EST-SSRs (Na = 5.444; Ho = 0.594; He = 0.613) have higher resolutions than microsatellite markers (Na = 2.000; Ho = 0.250–0.372; He = 0.432–0.456) [35] and genomic SSR markers (Na = 3.913; Ho = 0.468; He = 0.500) [1] in *A. tsaoko*. This phenomenon has also been reported in poplar [71]. PIC is one of the main indices used to evaluate locus polymorphisms, including high (PIC > 0.5), moderate (0.5 > PIC > 0.25), and low (PIC < 0.25) polymorphism [72]. Among the 72 accessions, the PIC values indicated a good informative level of these EST-SSRs, including 13 highly polymorphic (AM203, AM206, AM223, AM224, AM225, AM237, AM242, AM247, AM248, AM255, AM272, AM273, and AM278), four moderately polymorphic (AM207, AM213, AM218, and AM279), and only one low polymorphic (AM208) markers. EST-SSR markers exhibit much higher polymorphism than RAPD, ISSR, SRAP, and genomic SSR markers for *A. tsaoko* [1, 17, 34]. HWE analysis showed that 12 primer pairs (AM203, AM206, AM207, AM208, AM213, AM218, AM223, AM242, AM255, AM272, AM273, and AM279) were in accordance with HWE, while the remaining primer sets (AM224, AM225, AM237, AM247, AM248, and AM278) significantly deviated from HWE (*P* < 0.05). Taken together, the newly developed EST-SSRs are suitable for exploring the genetic diversity and relationships of *A. tsaoko*. These molecular markers will be effective tools in fingerprinting germplasm collections from different sources to guide germplasm evaluation and conservation in *A. tsaoko*. We also analyzed the characteristics of SSR loci within the terpenoid metabolism-related unigenes (Table S1). Next, we will develop molecular markers around these SSR loci, which is very important for gene mining and marker-assisted selection breeding for controlling important traits in *A. tsaoko*.

### Genetic diversity of the *A. tsaoko* population

Genetic diversity is an important indicator of the effective management and utilization of plant germplasm resources. The level of genetic diversity is often correlated with its range of distribution; the wide distribution range of plants usually shows higher levels of genetic diversity compared with narrowly distributed plants [73, 74]. However, while the known distribution of *A. tsaoko* is narrow, it retains relatively high levels of genetic diversity at the population level (PPB = 98.150%, I = 1.019, Ho = 0.594, and He = 0.560). Similar results have been reported for many other plant species, such as *Petunia secreta* [75], *Nouelia insignis* [76], and *Calanthe tsoongiana* [77]. In our study, Jingping and Lvchun counties, as possible places of origin of *A. tsaoko*, harbored the highest number of private alleles (PAr = 0.167), which indicates the presence of specific sequences or genes in JP and LVC populations [78]; this was also consistent with our previous findings [17]. The overall mean value of the fixation index was − 0.066, showing an excess of heterozygosity present in the *A. tsaoko* population; this result is in agreement with a previous study carried out with genomic SSR markers [1]. The pairwise F_ST_ analysis (average pairwise F_ST_ = 0.052) detected no significant genetic differentiation among six *A. tsaoko* populations. Correspondingly, the AMOVA results also showed that much more genetic variance exists among individuals within populations. High genetic diversity, excess heterozygotes, and low genetic differentiation are characteristics of outbreeding populations [79]. *A. tsaoko* is a perennial cross-pollinating plant pollinated by insects (native bumblebee), allowing allele exchange among individuals in the population [17]. In this study, the accessions did not cluster according to different sampling sites by UPGMA cluster, and the PCoA analysis further confirmed low genetic differentiation among populations.

Compared with bulk crops, the molecular markers available for Zingiberaceae are very limited. In our study, the ESR-SSR markers developed in *A. tsaoko* have high cross-species transferability (with an average transferability of 68.18%) with other Zingiberaceae species, and these markers can accurately classify different species, making them useful for increasing the number of available molecular markers of Zingiberaceae species.

## Conclusions

In this study, we reported the first transcriptome sequencing analysis of *A. tsaoko* using Illumina sequencing technology. The de novo assembly generated a total of 146,911 unigenes with an average length of 1527 bp and an N50 of 2002 bp. In total, 128,174 unigenes were successfully annotated to the NR (84.01%), NT (65.20%), KEGG (36.11%), Swiss-prot (65.84%), PFAM (60.97%), GO (61.09%), and KOG (24.92%) databases. 496 unigenes involved in the terpenoid biosynthesis pathway were identified. Based on the transcriptome assembly, 55,590 potential EST-SSRs were identified and characterized. Seventy-two *A. tsaoko* accessions using 18 novel polymorphic EST-SSR primers showed rich genetic diversity and low genetic differentiation among populations. The transcriptome data from this study will provide valuable resources for investigating gene functions, and the EST-SSR markers developed will provide a foundation for germplasm identification, genetic diversity assessment, and marker-assisted breeding in *A. tsaoko*.

## Methods

### Plant material

**Fig. 10:**
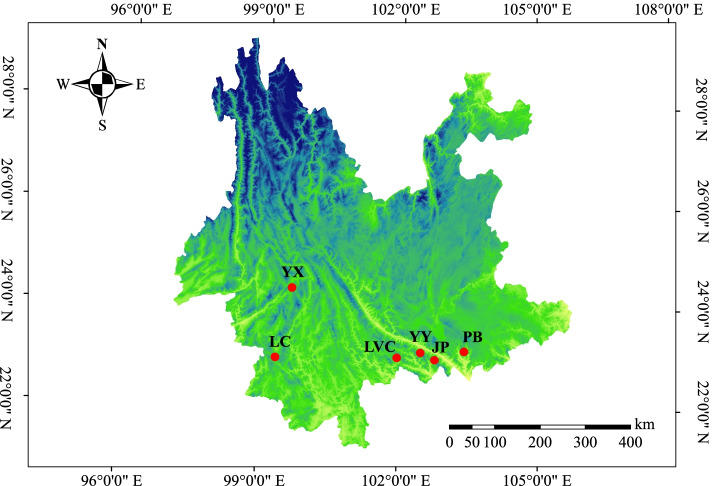
Geographic locations of the 6 populations of *A. tsaoko* included in this study

The transcriptome sequencing materials were collected from the young roots, stems, leaves, flowers, and fruits of 5-year-old *A. tsaoko* plants in Caoguoshan Village, Adebo Township, Jinping County, Honghe Prefecture, Yunnan Province, China (22°54′30.34″N, 103°13′16.39″E). To verify EST-SSR markers, young leaves from a total *of* 72 *A. tsaoko* accessions were collected from different sites covering the plant’s major distribution areas in China (Fig. 10; Table S2). For cross-species transferability analysis, 12 Zingiberaceae species were used, which consisted of two species in the genus *Amomum* (*A. tsaoko* and *A. villosum*), two species in *Kaempferia* (*K. galangal* and *K. rotunda*), two species in *Hedychium* (*H. flavum* and *H. coronarium*), and three species in *Curcuma* (*C. kwangsiensis*, *C. caesia*, and *C. phaeocaulis*) (Table S3). No approval or permission was required to collect these samples. All the species were identified by Professor Bingyue Lu (Honghe University). The genomic DNA was extracted from leaf tissues using a modified cetyltrimethylammonium bromide (CTAB) method [80].

### RNA isolation, library preparation, and transcriptome sequencing

Total RNA was extracted from three biological replicates (with two technical replicates per each biological replicate) from mixed samples of the five different organs using the RNeasy extraction kit (QIAGEN, Beijing, China). The integrity and purity of extracted RNA were measured by a NanoDrop spectrophotometer and agarose gel electrophoresis. After total RNA extraction, eukaryotic mRNA was enriched with Oligo (dT) beads. Next, fragmentation buffer was added to break mRNA into short fragments. Then, these mRNA fragments were used to synthesize first-strand cDNA with hexameric random primers. Subsequently, second-strand cDNA was synthesized by adding DNA polymerase I, RNase H, dNTPs, and buffer. The purified double-stranded cDNA was repaired at the end, dA-tailed fragments were added, and it was connected to the sequencing connector. After library inspection, the quality-qualified libraries were sequenced using the Illumina HiSeq platform (Novogene Biotech Co., Ltd., Beijing, China).

### De novo assembly and annotation

After sequencing, raw data were filtered, and high-quality clean data were obtained by removing the joint sequence and low-quality reads. High-quality reads were used for de novo assembly using Trinity software [81]. Finally, we obtained sequences that could not be extended on either end, which were considered unigenes. The resulting clean data were deposited in the Sequence Read Archive of National Center for Biotechnology Information (NCBI) (Bioproject no. PRJNA735890; Biosample no. SAMN19601270). The assembled *A. tsaoko* transcripts were compared with the NCBI non-redundant protein sequences (NR), NCBI non-redundant nucleotide sequences (NT), protein family (PFAM), eukaryotic clusters of orthologous groups (KOG), a manually annotated and reviewed protein sequence (SwissProt), and Kyoto Encyclopedia of Genes and Genomes (KEGG) databases using the BLASTX algorithm (significant thresholds of *E*-value < 10^− 5^), and Gene Ontology (GO) annotations were obtained using Blast2GO software (http://www.geneontology.org) based on NR annotations.

### Development and characterization of EST-SSRs

Based on the transcriptome sequencing data of *A. tsaoko*, MISA software (https://webblast.ipk-gatersleben.de/misa/) was used to search the SSR loci of *A. tsaoko*. The search criteria were as follows: mono- to hexa-nucleotide repeats ≥10, 6, 5, 5, 5, and 5; mono- to hexa-nucleotide repeat types are labeled P1, P2, P3, P4, P5, and P6. Primer3 (http://primer3.sourceforge.net/releases.php) was used to design SSR primers based on the detected sites.

### SSR analysis

Eighty pairs of EST-SSR primers were randomly synthesized, and four *A. tsaoko* germplasm resources with different fruit shapes (round type, oval type, long type, and shuttle type) were selected to verify the effectiveness and polymorphisms of the primers. The primers producing clear bands and obvious polymorphisms were subsequently used for genetic diversity assessments. The PCR reaction system, amplification program, and gel electrophoresis were as previously described [1].

### Data analysis

According to the polyacrylamide electrophoresis results, at the same migration location, a band is marked as “1,” no band is marked as “0,” and a missing band is indicated by “-”. Subsequently, 0/1 data were preprocessed using DataFormater software [82], which transformed SSR data into readable input files for NTSYS-pc and PowerMarker. The GenAlEx 6.501 program [83] was employed to calculate genetic diversity parameters, namely the observed number of alleles (Na), the effective number of alleles (Ne), Shannon’s information index (I), expected heterozygosity (He), observed heterozygosity (Ho), number of private alleles (PAr), and fixation index (F), which was used to assess Hardy–Weinberg genetic equilibrium. PowerMarker v3.25 [84] was used to estimate the polymorphism information content (PIC) of each EST-SSR primer pair. We estimated the frequencies of null alleles (FNA) using Micro-checker 2.2.3 [85]. The genetic differentiation between populations was carried out using analysis of molecular variance (AMOVA), pairwise F_ST,_ and pairwise Nei’s genetic distance in GenAlEx software. Cluster analysis (unweighted pair group method with arithmetic mean algorithm) and principal coordinate analysis (PCoA) were performed using NTSYS-pc software [86] and GenAlEx v6.5, respectively. Multiple amino acid sequence alignment and phylogenetic analysis was performed using DNAMAN 8.0 and MEGA version 5.2 software. The amino acid sequences of the 1,8-cineole synthase gene of *Arabidopsis thaliala* (GenBank accession no. NM_113483.5) and *Salvia officinalis* (GenBank accession no. AF051899.1) were downloaded from the NCBI (http://www.ncbi.nlm.nih.gov/). The amino acid sequences of four monoterpene synthase genes (*AvTPS1*, *AvTPS2*, *AvTPS3*, and *AvTPS4*) of *A. villosum* were obtained from the published literature [87].

## Supplementary Information

**Additional file 1: Table S1.** Analysis of SSR loci of terpenoid metabolic pathway-related unigenes in *A. tsaoko*. **Table S2.** Populations of *A. tsaoko* from different locations in the study. **Table S3.** Sampling location information of 12 Zingiberaceae species.

**Additional file 2: Fig. S1.** The original image of electrophoretic using AM242, AM272, AM273, and AM278 primer pairs.

## Data Availability

The original sequencing data generated in the study have been deposited into the National Center for Biotechnology Information (NCBI) Sequence Read Archive (SRA) database (https://www.ncbi.nlm.nih.gov/bioproject/?term=PRJNA735890). The EST sequences in this study have been submitted to GenBank with accession numbers OM468473–OM468552 (https://www.ncbi.nlm.nih.gov/genbank/). Other datasets supporting the conclusions of this article are included within the article.

## Abbreviations

EST-SSR: Expressed sequence tag-simple sequence repeat
AMOVA: Analysis of molecular variance
TCM: Traditional Chinese medicine
COVID: Coronavirus disease
IUCN: International union for conservation of nature
RFLP: Restriction fragment length polymorphism
RAPD: Random amplified polymorphic DNA
ISSR: Inter-simple sequence repeat
SSR: Simple sequence repeat
SNP: Single nucleotide polymorphism
QTL: Quantitative trait locus
CTAB: Cetyltrimethylammonium bromide
NR: NCBI non-redundant protein sequences
NT: NCBI non-redundant nucleotide sequences
PFAM: Protein family
KOG: Eukaryotic clusters of orthologous groups
SwissProt: A manually annotated and reviewed protein sequence
KEGG: Kyoto encyclopedia of genes and genomes
GO: Gene ontology
Na: Observed number of alleles
Ne: Effective number of alleles
I: Shannon’s information index
He: Expected heterozygosity
Ho: Observed heterozygosity
PAr: Number of private alleles
F: Fixation index
FNA: Frequencies of null alleles
UPGMA: Unweighted pair-group method with arithmetic means
PCoA: Principal coordinate analysis
HWE: Hardy–Weinberg genetic equilibrium
