# Supplementary material for: De novo transcriptome assembly, gene annotation, and EST-SSR marker development of an important medicinal and edible crop, Amomum tsaoko (Zingiberaceae)
Source: BMC Plant Biol. 2022 Sep 29;22:467. doi: 10.1186/s12870-022-03827-y (PMC9519402; doi:10.1186/s12870-022-03827-y)
Supplement: Supplementary file 1 — Additional file 1: Table S1. Analysis of SSR loci of terpenoid metabolic pathway-related unigenes in A. tsaoko. Table S2. Populations of A. tsaoko from different locations in the study. Table S3. Sampling location information of 12 Zingiberaceae species. [file 12870_2022_3827_MOESM1_ESM.docx]

**Table S1** Analysis of SSR loci of terpenoid metabolic pathway-related unigenes in *A. tsaoko*

| Pathway | KO ID | KO Name | KO Description | SSR | Unigene ID |
| --- | --- | --- | --- | --- | --- |
| Terpenoid backbone biosynthesis | K01662 | dxs | 1-deoxy-D-xylulose-5-phosphate synthase | (A)10 | Cluster-26586.50325, Cluster-26586.46511, Cluster-26586.46513, Cluster-26586.52986, Cluster-26586.56940 |
|  |  |  |  | (T)10 | Cluster-26586.77374 |
|  |  |  |  | (A)13 | Cluster-26586.55105 |
|  |  |  |  | (GA)6 | Cluster-26586.47899 |
|  | K05954 | FNTB | protein farnesyltransferase subunit beta | (A)10 | Cluster-26586.74828, Cluster-26586.13461 |
|  |  |  |  | (T)11 | Cluster-26586.67974, Cluster-26586.37817, Cluster-26586.22589, Cluster-26586.13460 |
|  |  |  |  | (TA)7 | Cluster-26586.22301 |
|  | K00938 | E2.7.4.2, mvaK2 | phosphomevalonate kinase | (T)10 | Cluster-26586.30046 |
|  | K00626 | E2.3.1.9, atoB | acetyl-CoA C-acetyltransferase | (T)10 | Cluster-26586.69090, Cluster-26586.46812, Cluster-26586.60661, Cluster-26586.46803 |
|  | K00787 | FDPS | farnesyl diphosphate synthase | (T)10 | Cluster-26586.40079 |
|  |  |  |  | (T)15gcttttgggacctttcttgagtgcatggctcttaagaa(T)10 | Cluster-26586.62395 |
|  | K11778 | DHDDS, RER2, SRT1 | ditrans,polycis-polyprenyl diphosphate synthase | (CGC)7cgtcgccgccgt(CGC)6 | Cluster-26586.33985 |
|  |  |  |  | (A)10 | Cluster-26586.1506 |
|  | K00099 | dxr | 1-deoxy-D-xylulose-5-phosphate reductoisomerase | (T)14 | Cluster-26586.89044, Cluster-26586.89045, Cluster-26586.89046, Cluster-25768.0, Cluster-26586.12307 |
|  | K06013 | STE24 | STE24 endopeptidase | (A)13 | Cluster-26586.62245 |
|  |  |  |  | (A)11 | Cluster-26586.96162 |
|  | K15891 | FLDH | farnesol dehydrogenase | (A)10 | Cluster-25693.0, Cluster-26741.0 |
|  | K15892 | FOLK | farnesol kinase | (TCC)5 | Cluster-23484.0 |
|  | K10960 | chlP, bchP | geranylgeranyl reductase | (A)18 | Cluster-26586.73059, Cluster-26586.59159, Cluster-26586.56772, Cluster-26586.56771, Cluster-26586.30132, Cluster-26586.65277,  Cluster-26586.57443 |
|  |  |  |  | (GA)6 | Cluster-26586.73059, Cluster-26586.56772, Cluster-26586.56771, Cluster-26586.30132, Cluster-26586.65277, Cluster-26586.57443 |
|  |  |  |  | (AGG)6 | Cluster-26586.66130 |
|  | K15095 | E1.1.1.208 | (+)-neomenthol dehydrogenase | (A)14 | Cluster-26586.48850, Cluster-26586.48851, Cluster-26586.48852, Cluster-26586.48849, Cluster-26586.48848, Cluster-26586.69915,  Cluster-26586.33757, Cluster-26586.65008, Cluster-26586.65004, Cluster-26586.65003 |
|  | K01823 | idi, IDI | isopentenyl-diphosphate delta-isomerase | (C)33 | Cluster-26586.93801, Cluster-26586.93797 |
|  |  |  |  | (C)79 | Cluster-26586.88716, Cluster-26586.93796 |
|  | K03526 | gcpE, ispG | (E)-4-hydroxy-3-methylbut-2-enyl-diphosphate synthase | (T)10 | Cluster-26586.65877, Cluster-26586.94572 |
|  |  |  |  | (CT)14 | Cluster-26586.33007 |
|  |  |  |  | (TC)15 | Cluster-26586.46449 |
|  |  |  |  | (TC)13 | Cluster-26586.46448 |
|  | K13789 | GGPS | geranylgeranyl diphosphate synthase, type II | (G)10 | Cluster-26586.39364 |
|  |  |  |  | (GATC)5 | Cluster-26586.13749 |
|  |  |  |  | (CT)11 | Cluster-26586.53919, Cluster-26586.53918 |
| Monoterpenoid biosynthesis | K15086 | TPS14 | (3S)-linalool synthase | (A)10 | Cluster-18694.0 |
|  | K15095 | E1.1.1.208 | (+)-neomenthol dehydrogenase | (A)14 | Cluster-26586.33757, Cluster-26586.48848, Cluster-26586.48849, Cluster-26586.48850, Cluster-26586.48851, Cluster-26586.48852,  Cluster-26586.65003, Cluster-26586.65004, Cluster-26586.65008, Cluster-26586.69915 |
| Diterpenoid biosynthesis | K04125 | E1.14.11.13 | gibberellin 2-oxidase | (TTC)5 | Cluster-31775.0, Cluster-26586.11361 |
|  |  |  |  | (AGG)6 | Cluster-26586.32064 |
|  |  |  |  | (A)10 | Cluster-2985.0, Cluster-27208.0 |
|  | K04120 | E5.5.1.13 | ent-copalyl diphosphate synthase | (TC)6 | Cluster-26586.21337, Cluster-26586.56838 |
|  |  |  |  | (T)12gg(A)13 | Cluster-26586.89264, Cluster-26586.40317 |
|  |  |  |  | (TA)11 | Cluster-26586.7319, Cluster-26586.4187, Cluster-26586.101416 |
|  | K04123 | KAO | ent-kaurenoic acid hydroxylase | (A)10 | Cluster-26586.55512, Cluster-26586.55514, Cluster-2294.0 |
|  |  |  |  | (TA)8 | Cluster-5322.1, Cluster-5322.2, Cluster-9276.0 |
|  | K05282 | E1.14.11.12 | gibberellin 20-oxidase | (AG)10 | Cluster-9027.0, Cluster-26586.28318, Cluster-22145.0 |
|  |  |  |  | (AAAACA)5 | Cluster-9027.0, Cluster-12516.1, Cluster-8671.0 |
|  |  |  |  | (AAAACA)6 | Cluster-12516.3 |
|  |  |  |  | (GGC)5 | Cluster-26586.9933, Cluster-26586.99381 |
|  |  |  |  | (TA)6ac(A)14 | Cluster-26586.9933, Cluster-26586.28318 |
|  |  |  |  | (AGA)5 | Cluster-26586.65925, Cluster-26586.95987 |
| Sesquiterpenoid and triterpenoid biosynthesis | K15891 | FLDH | farnesol dehydrogenase | (A)10 | Cluster-25693.0, Cluster-26741.0 |
|  | K15472 | CYP71D55 | premnaspirodiene oxygenase | (AT)6 | Cluster-26586.65385 |
|  | K00511 | SQLE, ERG1 | squalene monooxygenase | (T)10 | Cluster-26586.43355 |
|  | K00801 | FDFT1 | farnesyl-diphosphate farnesyltransferase | (TC)7 | Cluster-26586.79933, Cluster-26586.79931 |

**Table S2** Populations of *A. tsaoko* from different locations in the study

| Population | No. of samples | Latitude (N) | Longitude (E) | Altitude (m) | Location |
| --- | --- | --- | --- | --- | --- |
| PB | 12 | 23°2'24" | 103°31'48" | 1721 | Pingbian, Yunnan |
| JP | 12 | 22°54'36" | 103°13'12" | 1665 | Jingping, Yunnan |
| YY | 12 | 23°3'0" | 102°55'12" | 2108 | Yuanyang, Yunnan |
| LVC | 12 | 22°53'24" | 102°24'43" | 1880 | Lvchun, Yunnan |
| YX | 12 | 24°17'24" | 100°6'36" | 1811 | Yunxian, Yunnan |
| LC | 12 | 22°54'36" | 99°49'12" | 1924 | Lancang, Yunnan |

**Table S3** Sampling location information of 12 Zingiberaceae species

| Species | Latitude (E) | Longitude (N) | Altitude (m) | Location |
| --- | --- | --- | --- | --- |
| *Amomum tsaoko* | 22°44′23.93″ | 103°12′51.54″ | 1381 | Jinping, Yunnan |
| *Amomum villosum* | 22°44′23.93″ | 103°12′51.54″ | 1381 | Jinping, Yunnan |
| *Alpinia coriandriodora* | 23°16′3.15″ | 105°49′35.62″ | 804 | Napo, Guangxi |
| *Alpinia oxyphylla* | 22°03′45.98″ | 111°57′46.38″ | 12 | Yangjiang, Guangdong |
| *Alpinia zerumbet* | 23°21′2.66″ | 103°25′42.12″ | 1309 | Mengzi, Yunnan |
| *Kaempferia galanga* | 23°21′2.66″ | 103°25′42.12″ | 1309 | Mengzi, Yunnan |
| *Kaempferia rotunda* | 22°44′23.93″ | 103°12′51.54″ | 1381 | Jinping, Yunnan |
| *Hedychium flavum* | 22°44′23.93″ | 103°12′51.54″ | 1381 | Jinping, Yunnan |
| *Hedychium coronarium* | 22°44′23.93″ | 103°12′51.54″ | 1381 | Jinping, Yunnan |
| *Curcuma kwangsiensis* | 22°57′57.65″ | 103°12′45.69″ | 1310 | Jinping, Yunnan |
| *Curcuma caesia* | 22°57′57.65″ | 103°12′45.69″ | 1310 | Jinping, Yunnan |
| *Curcuma phaeocaulis* | 22°57′57.65″ | 103°12′45.69″ | 1310 | Jinping, Yunnan |
