# Supplementary material for: De novo transcriptome assembly, gene annotation, and EST-SSR marker development of an important medicinal and edible crop, Amomum tsaoko (Zingiberaceae)
Source: BMC Plant Biol. 2022 Sep 29;22:467. doi: 10.1186/s12870-022-03827-y (PMC9519402; doi:10.1186/s12870-022-03827-y)
Supplement: Supplementary file 2 — Additional file 2: Fig. S1. The original image of electrophoretic using AM242, AM272, AM273, and AM278 primer pairs. [file 12870_2022_3827_MOESM2_ESM.pdf]

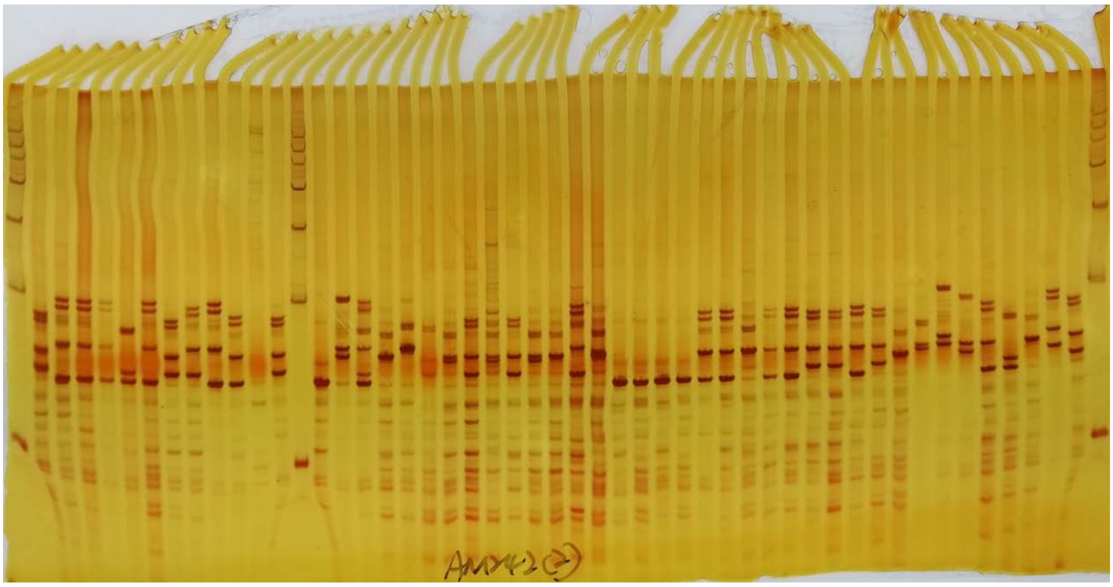

The original image of electrophoretic using AM242 primer pair

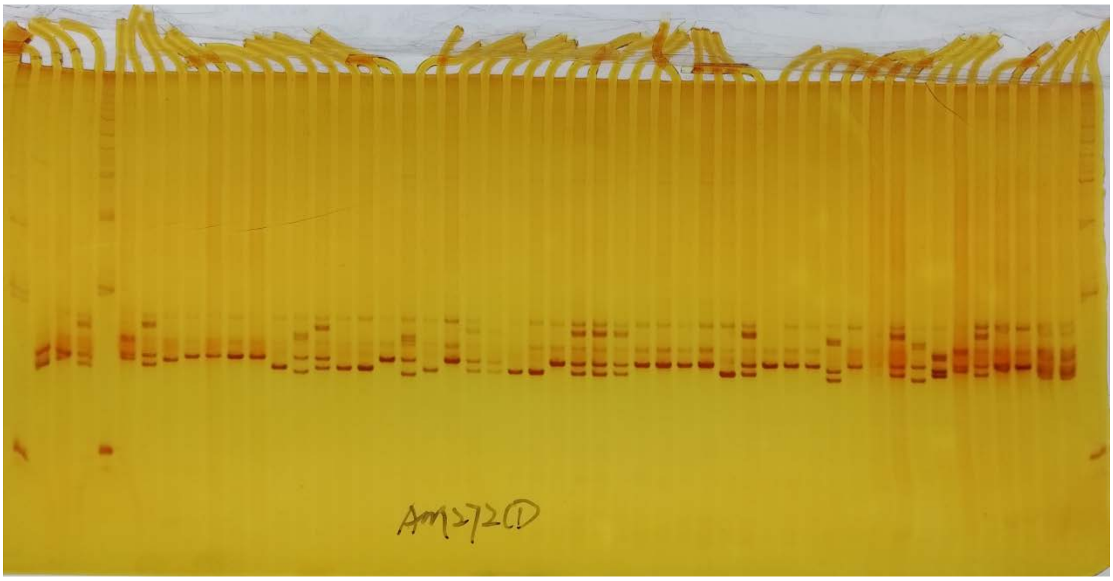

The original image of electrophoretic using AM272 primer pair

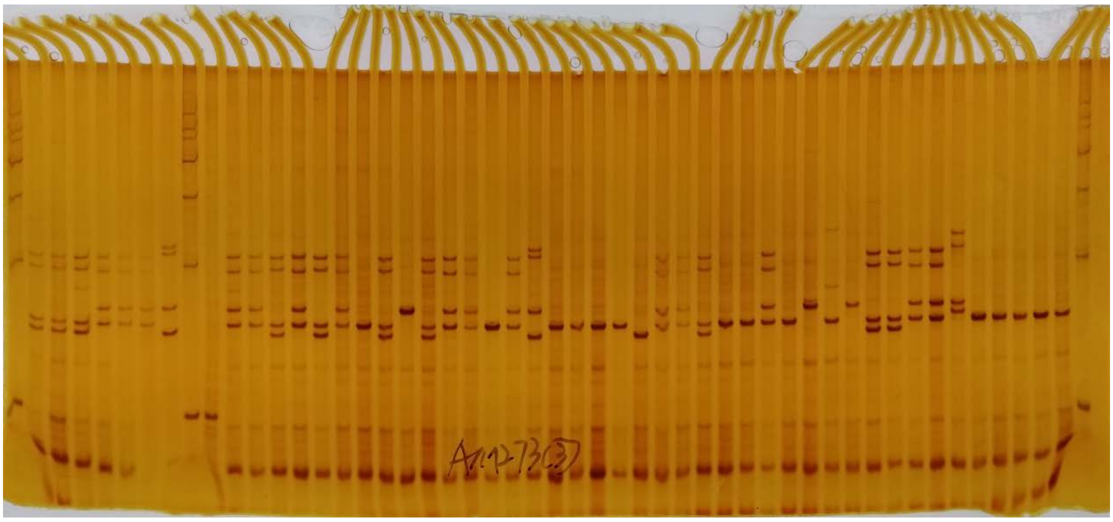

The original image of electrophoretic using AM273 primer pair

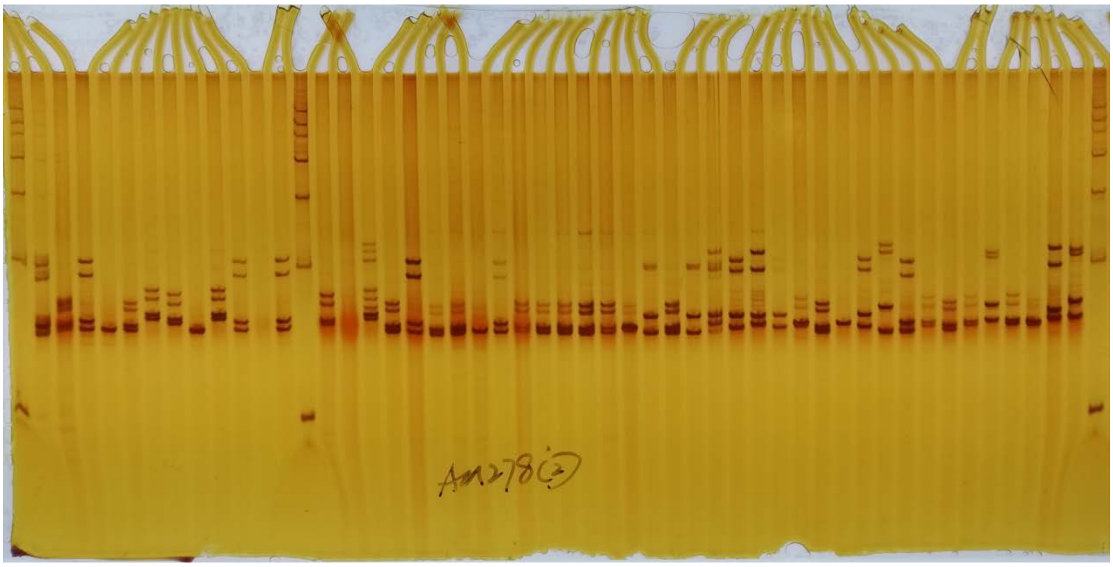

The original image of electrophoretic using AM278 primer pair
